# Outcome of patients older than 85 years hospitalized in a neurology unit

**DOI:** 10.1007/s40520-023-02468-x

**Published:** 2023-06-18

**Authors:** Giacomo Querzola, Andrea Bellomo, Emilia Salvadori, Leonardo Pantoni

**Affiliations:** 1 Department of Biomedical and Clinical Sciences, University of Milan, Milan, Italy; 2 NEUROFARBA Department, Neuroscience Section, University of Florence, Florence, Italy; 3 Neurology Unit, Luigi Sacco University Hospital, Milan, Italy

**Keywords:** Hospitalization, Outcome, Neurological diseases, Elderly, Frailty, Aging

## Abstract

**Background:**

Advanced age is a major determinant of mortality and poor outcome at any level. In hospitalized patients, advanced age is a major issue in terms of prognosis, resource use, and therapeutic choices.

**Aims:**

We aimed at assessing the 1 year outcome of elderly patients admitted to a neurology unit for various acute conditions.

**Methods:**

Consecutive patients admitted to a neurology unit were enrolled and followed-up at 3, 6, and 12 months with structured phone interviews gathering information about mortality, disability, hospital readmissions, and place of residency. Inclusion criteria were age ≥ 85 years, availability of written consent and phone contact; no exclusion criteria were applied.

**Results:**

Over a period of 16 months, 131 patients (88.8 ± 3.3, 92 females, 39 males) were admitted. The pre-hospitalization modified Rankin Scale (mRS) median (IQR) score, obtainable in 125 patients, was 2 (0, 3) and > 3 in 28/125 (22.4%) patients. Fifty-eight (46.8%) patients had pre-existing dementia (this information was missing for one patient). Eleven patients died during hospitalization. Of the 120 discharged patients, 60 (50%) were alive at 12 months, 41 died during follow-up (34.2%), and 19 (15.8%) were lost. At 12 months, out of the 60 alive patients, 29 (48.3%) had a mRS > 3. We did not detect predictors of 12-month survival. Predictors of 12-month worsening of functional status were pre-hospitalization mRS, pre-existing cognitive impairment, and male sex.

**Conclusions:**

One-year mortality of elderly patients admitted to a neurology unit is extremely high. After one year, less than one fourth of elderly patients hospitalised for an acute neurological disease are left with only no-to-moderate disability.

**Supplementary Information:**

The online version contains supplementary material available at 10.1007/s40520-023-02468-x.

## Introduction

Aging of the population is a major issue. The World Health Organization estimates that in the next 30 years, the world's population over 60 years will pass from 12 to 22%, with consequences for all countries that will have to face major health and social challenges [1]. Consequently, also the mean age of hospitalised patients is increasing. Although a few data about elderly patients hospitalized in other specialty wards are available (intensive care unit, internal medicine, rehabilitation, post-acute), there is a lack of studies on elderly patients admitted to a neurological ward [2, 3].

Many neurological diseases, such as cerebrovascular and neurodegenerative ones, are age-related and many of them may lead to hospitalization with increasing direct costs. Neurological diseases are also associated with high rates of disability and mortality. It is therefore relevant to understand the outcome of the oldest-old patients hospitalized for neurological diseases and to outline possible predictors of outcome in this very frail population to better assess and plan the health investments and the interventions during hospitalization and thereafter.

Considering the paucity of data about this particular group of patients, we aimed to assess: (1) the prognosis of hospitalized oldest-old neurological patients in terms of mortality and functional outcome; and (2) the factors associated with their functional autonomy and survival.

## Methods

This was a prospective study on ≥ 85-year-old, consecutive patients admitted to the neurological ward of the “Luigi Sacco” hospital in Milan from January 1, 2019 to May 31, 2020. The COVID-19 pandemic outbreak led to a marked reduction in admissions to our ward, and thus to the decision to stop the study on May 31, 2020.

The study was performed in accordance with the 1964 Declaration of Helsinki and its later amendments. Informed consent was obtained by patients or caregivers.

### Patients sample

Inclusion criteria were: (1) age ≥ 85 years; (2) neurology unit admission from emergency room; (3) written consent given by the patient or caregiver; (4) availability of a phone contact. No exclusion criterion was applied.

### Data collection during hospitalization

Within 48 h of admission, we collected information about: (1) age; (2) sex; (3) place of provenance (home, rehabilitation center, nursing home, other medical ward); (4) social and family environment (living alone, with autonomous/non-autonomous partner, with other relatives, assisted by an attendant); (5) cause of hospitalization; (6) associated diseases; (7) dementia, defined as Clinical Dementia Rating (CDR) ≥ 1 [4]; (8) functional status before the current hospitalization assessed with the modified Rankin Scale (mRS) [5]; (9) stroke severity evaluated by means of the National Institutes of Health Stroke Scale (NIHSS) at admission.

At discharge, we gathered data about: (1) length of hospitalization in days; (2) discharge diagnosis; (3) discharge destination.

### Follow-up

Phone interviews were made at 3 months (± 5 days), 6 months (± 10 days), and 12 months (± 20 days) after discharge, inquiring the caregiver about: (1) current living place; (2) new hospitalization; (3) functional status (assessed with mRS); (4) possible COVID-19 related events (this question was added after January 2020).

Patients for whom no phone answer was obtained on five phone calls in 3 or more different days were considered as lost at follow-up.

### Statistical analysis

The functional study outcome, as measured by means of the modified Rankin Scale (mRS), was dichotomized as none-to-moderate degree of disability (mRS ≤ 3) vs. severe disability (3 < mRS < 6) at the follow-up visits. Furthermore, a comorbidity sum score was computed taking into account the presence of each of the following conditions: cognitive impairment, previous stroke, hypertension, diabetes, cancer, heart failure, ischemic heart disease, previous surgery (general or neurosurgical), other neurological, cardiologic, orthopedic, oculistic, pulmonary, gastroenterological, hematologic, endocrine, otorhinolaryngologic, psychiatric, nephrological, rheumatologic, vascular, and dermatologic diseases. The comorbidity sum score ranged from 0 (no condition present) to 23 (all conditions present) [6].

Descriptive statistics (frequencies and percentages, median and interquartile range, or means and standard deviations) were used to illustrate the total sample characteristics.

Univariate statistical analyses (independent sample t test, Wilcoxon–Mann–Whitney *U* test, Pearson’s chi-squared test) were used to compare alive and dead patients in terms of demographics, presence of cognitive impairment, pre-hospitalization functional status, comorbidities, length of hospitalization, discharge diagnosis and destination, and emergency room access or hospitalization at each time point (3, 6, and 12 months’ follow-up visits). The same models of analyses were used to compare patients presenting a none-to-moderate degree of disability and those with a severe disability at the follow-up visits.

Multivariate logistic regression models were used to evaluate the interaction among the characteristics that clinically influence the survival and functional outcomes and resulted significantly associated in univariate analyses. All multivariate logistic regressions used a full model adjusted for age and pre-hospitalization mRS and odds ratio (OR) with 95% confidence intervals (95% CI) were reported.

To explore the influence of the heterogeneity of discharge diagnoses and of the pre-morbid functional status, the univariate and multivariate models of analyses were repeated in two subgroups analyses: (1) limited to patients with a discharge diagnosis of cerebrovascular event, and (2) limited to patients with a pre-hospitalization mRS ≤ 3.

All analyses were done using the SPSS software version 27, and a 0.05 significance threshold was applied.

## Results

### Characteristics of the participants

**Fig. 1:**
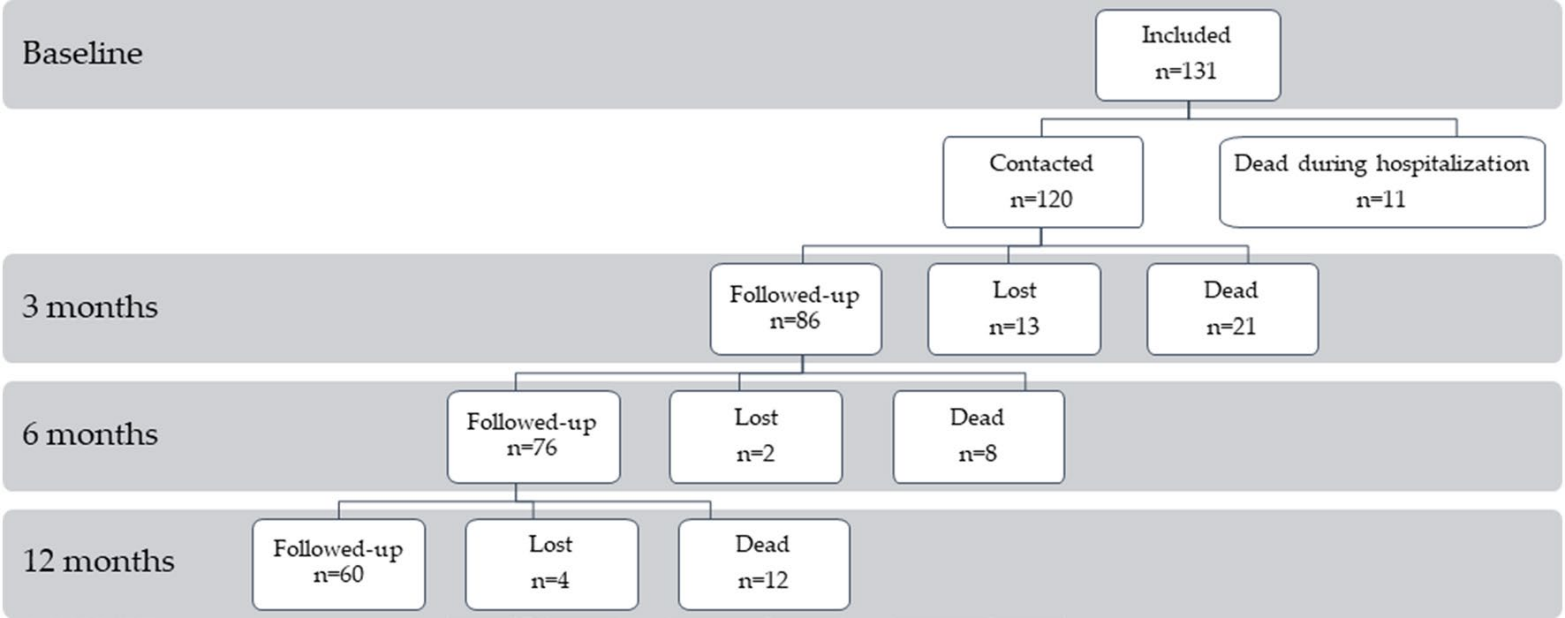
Flow diagram showing patients’ attrition from the inclusion phase to follow-up assessments (no exclusion criterion was applied)

Over the study period, 131 patients (mean age ± SD 88.84 ± 3.29 years; 92 females, 39 males) were admitted to the neurology ward (Fig. 1). Median (IQR) duration of hospital stay was 9 (5, 14) days.

In 92/131 patients (70.2%) the cause of hospitalization was an acute cerebrovascular event. Less frequent causes of hospitalization were gait disturbance or falls (n = 13), seizure (n = 8), acute confusional state or vigilance disturbance (n = 5), headache (n = 2), visual impairment (n = 2). Other causes accounted for 6.9% (n = 9).

Data on living condition and referral were available in 130 patients. Out of 130 patients, 125 (96%) were by living home, and five were transferred from other hospital wards. Among the 125 patients that lived at home, 50 (40%) were living alone, 27 (22%) lived with a partner, 21 (17%) with other relatives, and 27 (22%) were assisted by a full-time caregiver before hospitalization.

The pre-hospitalization mRS score was obtainable in 125 patients and the median (IQR) value was 2 (0, 3). mRS was > 3 in 28/125 (22.4%) patients. Fifty-eight (46.8%) patients had pre-existing dementia (defined as CDR > 1) (information about antecedent cognitive status was not obtainable in one patient). Comorbidities are reported in Table 1. A previous stroke was reported in 18/125 (14.4%).

**Table 1:** Comorbidities at hospital admission

Comorbidities	
Hypertension	75.4% (95/126)
Previous surgery	70.6% (89/126)
Cardiologic	49.6% (62/125)
Orthopedic	49.2% (62/126)
Dementia (CDR > 1)	46.8% (58/124)
Neurologic (excluding stroke)	37.3% (47/126)
Vascular	27% (34/126)
Gastroenterological	26.2% (33/126)
Nephrological	25.4% (32/126)
Endocrine	22.2% (28/126)
Diabetes mellitus	20.0% (25/125)
Ophthalmic	19.8% (25/126)
Hematologic	17.5% (22/126)
Previous stroke	14.4% (18/125)
Otorhinolaryngologic	14.4% (18/125)
Pneumological	12.7% (16/126)
Psychiatric	9.5% (12/126)
Rheumatologic	7.1% (9/126)
Active cancer	6.4% (8/125)
Dermatologic	4.8% (6/126)
Previous neurosurgery	4.8% (6/126)
Comorbidity (sum score)	5.7 ± 2.8 (range min–max 0–12)

*CDR*: Clinical Dementia Rating scale

Eleven patients (8.4%) died during hospitalization (Fig. 1). Discharge diagnoses are reported in Table 2. The three most frequent discharge diagnoses were ischemic (67/120, 55.8%) or hemorrhagic stroke (9/120, 7.5%) and traumatic brain injury (8/120, 6.7%). Among the 72 patients with a discharge diagnosis of a cerebrovascular event, the mean NIHSS score was 8.4 ± 7.6. 70% of patients were discharged home, 6/120 (5.0%) were transferred to hospice, 4/120 (3%) to other wards, 23/120 (19.2%) were transferred to rehabilitation ward, and 6/120 (5.0%) to a nursing home.

**Table 2:** Discharge diagnosis

Discharge diagnosis	
Ischemic stroke	55.8% (67/120)
Hemorragic stroke	7.5% (9/120)
Traumatic brain injury	6.7% (8/120)
Seizure	5.0% (6/120)
TIA	4.2% (5/120)
Brain neoplastic lesion	2.5% (3/120)
Infectious encephalitis	1.7% (2/120)
Dizziness	1.7% (2/120)
Acute confusional state	1.7% (2/120)
Myelitis	1.7% (2/120)
Parkinson’s complication	0.8% (1/120)
Behavioral disturbance	0.8% (1/120)
Giant cell arteritis	0.8% (1/120)
Peripheral neuropathy	0.8% (1/120)
Chronic vascular encephalopathy	0.8% (1/120)
Cranial multineuropathy	0.8% (1/120)
Vertebral abscess	0.8% (1/120)
Subdural hygroma	0.8% (1/120)
Myasthenia gravis	0.8% (1/120)
Paralytic ileus and urinary retention	0.8% (1/120)
Cerebral amyloid angiopathy	0.8% (1/120)
Toxic encephalopathy	0.8% (1/120)
Normal pressure hydrocephalus	0.8% (1/120)
Acute anxiety episode	0.8% (1/120)

### Outcome

At the 3-month phone follow-up, we obtained information on 107/120 patients (89.1%) of whom 28% were males (Table 3). Eighty-six patients were alive, and 21 were dead (Fig. 1). 68/86 (79.0%) patients were living at home, but only 39% were living alone. mRS was ≤ 3 in 58% (50/86) of patients. In 26/86 patients (30%) a new emergency room access or hospitalization was recorded. COVID-19 infection was reported in only one patient and no death was correlated to COVID-19 infection.

**Table 3:** Characteristics of patients seen at the follow-up at 3, 6 and 12 months

	3 months follow-up		6 months follow-up		12 months follow-up	
	n = 107		n = 84		n = 72	
At baseline						
Age	107	88.7 ± 3.1	84	88.4 ± 2.8	72	88.5 ± 2.7
Sex (male)	107	30 (28%)	84	24 (29%)	72	21 (29%)
Coming from home	107	104 (97%)	84	81 (96%)	72	70 (97%)
Living condition (alone)	104	41 (39%)	83	34 (41%)	72	28 (39%)
Cognitive impairment	102	46 (45%)	79	33 (42%)	68	29 (43%)
Pre-hospitalization mRS	104	2 (0, 3)	82	2 (0, 3)	70	1.5 (0, 3)
Length of hospitalization	105	9 (4.5, 13.5)	84	8 (4.25, 12)	72	7 (4, 12)
Discharge diagnosis (cerebrovascular event)	107	72 (67%)	84	57 (68%)	72	49 (68%)
Comorbidity (sum score)	107	5.8 ± 2.7	84	5.7 ± 2.8	72	5.5 ± 2.7
After discharge						
Discharge destination (home)	103	72 (70%)	84	60 (71%)	72	52 (72%)
ER access or hospitalization	86	26 (30%)	76	28 (37%)	60	18 (30%)

*mRS* modified Rankin scale, *ER* emergency room

At the 6-month follow-up, we obtained phone information from 84 patients, 29% were male (Table 3). Seventy-six patients were alive, and eight were dead (Fig. 1). 67/76 (88.1%) of patients were still living at home, but only 41% were living alone. mRS was ≤ 3 in 60.5% of patients. 28/76 patients (37%) has had a new emergency room access or hospitalization. Three patients referred a COVID-19 infection in the previous three months, in one case leading to hospitalization.

At the 12-month follow-up, we assessed the status of 72 patients, 29% male (Fig. 1 and Table 3). 60/72 (83.3%) patients were still alive and 97% lived at home, alone in 39% of the cases. mRS was ≤ 3 in 43/60 (71.6%). 18/60 patients (30%) has had a new emergency room access or hospitalization. Two patients had a COVID19 infection in the previous 6 months, in both cases leading to hospitalization. None of the admitted patients during the outbreak period (February–May 2020) was COVID -19 positive.

Considering the 120 discharged patients of the sample initially admitted to the neurology ward, 60 (50%) were still alive at 12 months, 41 died during follow-up (34.2%), and 19 (15.8%) were lost. At 12 months from the discharge, out of the 60 alive patients, 43 (72%) had a mRS ≤ 3.

### Potential outcome predictors

Pre-hospitalization mRS was associated with both outcomes at 3 months (survival and mRS ≤ 3, respectively Wilcoxon-Mann–Whitney U test p = 0.013 and p = 0.001, Tables 4 and 5), and the same result was confirmed in subgroup analyses limited to stroke patients (Wilcoxon-Mann–Whitney U test p = 0.031 and p = 0.001, supplementary tables 2 and 3). In univariate analysis, living condition (alone), cognitive impairment (absent), lower comorbidity sum score, shorter length of hospitalization, and discharge destination (home) had a statistically significant association with mRS ≤ 3 at 3 months (Table 4). Living condition, comorbidity sum score, and discharge destination were associated with the functional outcome at 3 months also in subgroup analyses limited to patients with stroke or with a pre-hospitalization mRS ≤ 3 (supplementary tables 2 and 5). At 6 months, pre-hospitalization mRS was associated only with the functional outcome (whole sample p = 0.009, stroke patients p = 0.010), length of hospitalization with the survival outcome (whole sample p = 0.026, patients with pre-hospitalization mRS ≤ 3 p = 0.017), while the comorbidity sum of score was associated with both outcomes in the whole sample (survival p = 0.013, functional p = 0.017) and in stroke patients (survival p = 0.001, functional p = 0.012), and only with the functional outcome in patients with pre-hospitalization mRS ≤ 3 (p = 0.022) (Tables 4 and 5, supplementary tables 2, 3, 5 and 6). Finally, factors associated with the functional outcome at 12 months were pre-hospitalization mRS (whole sample p = 0.004, stroke patients p = 0.017), pre-existing cognitive impairment (whole sample p = 0.015, stroke patients p = 0.011, patients with pre-hospitalization mRS ≤ 3 p = 0.026), and male sex (whole sample p = 0.032). We did not detect factors associated with the 12-month survival (Table 5, supplementary tables 3 and 6).

**Table 4:** Factors associated with the functional outcome at 3, 6 and 12 months

	3 months follow-up				6 months follow-up				12 months follow-up			
	mRS ≤ 3	3 < mRS < 6	p	Logistic regression*	mRS ≤ 3	3 < mRS < 6	p	Logistic regression*	mRS ≤ 3	3 < mRS < 6	p	Logistic regression*
	n = 50	n = 36		OR (95%CI)	n = 46	n = 30		OR (95%CI)	n = 43	n = 17		OR (95%CI)
At baseline												
Age	88.1 ± 2.6	88.75 ± 3.1	0.327^+^	1.07 (0.85–1.34)	88.0 ± 2.5	89.1 ± 2.9	0.080^+^	1.13 (0.91–1.39)	88.2 ± 2.4	89 ± 3.1	0.297^+^	1.07 (0.82–1.39)
Sex (male)	16 (32%)	10 (28%)	0.674^#^		15 (33%)	7 (23%)	0.383^#^		**14 (33%)**	**1 (6%)**	**0.032**^#^	
Coming from home	49 (98%)	34 (94%)	0.375^#^		45 (98%)	29 (97%)	0.758^#^		42 (98%)	16 (94%)	0.489^#^	
Living condition (alone)	**28 (56%)**	**7 (19%)**	**0.001**^#^	**0.30 (0.09-0.99)**	23 (50%)	19 (63%)	0.155^#^	0.52 (0.16–1.66)	20 (46%)	6 (35%)	0.429^#^	1.43 (0.33–6.09)
Cognitive impairment	**15 (33%)**	**19 (54%)**	**0.050**^#^	1.67 (0.47–5.90)	15 (36%)	16 (55%)	0.104^#^	1.31 (0.39–4.46)	**12 (30%)**	**11 (65%)**	**0.015**^#^	0.41 (0.09–1.67)
Pre-hospitalization mRS	**1 (0, 2)**	**3 (1, 4)**	**0.001°**	**1.83 (1.22–2.74)**	**1 (0, 2)**	**3 (0, 4)**	**0.009°**	**1.57 (1.06–2.34)**	**1 (0, 2)**	**3 (1.5, 4)**	**0.004°**	**1.62 (1.05–2.72)**
Length of hospitalization	**7 (4, 11.25)**	**10 (6, 16)**	**0.006°**	1.12 (1.00–1.26)	7 (4, 11.25)	8.5 (5, 13.25)	0.178°	1.02 (0.94–1.12)	6 (4, 11)	8 (6, 13)	0.139°	1.02 (0.93–1.11)
Discharge diagnosis (cerebrovascular event)	31 (62%)	28 (78%)	0.120^#^		28 (61%)	24 (80%)	0.079^#^		26 (60%)	14 (82%)	0.105^#^	
Comorbidity (sum score)	**4.9 ± 2.4**	**6.8 ± 2.9**	**0.002**^+^	1.23 (0.97–1.55)	**4.8 ± 2.3**	**6.4 ± 3.1**	**0.017**^+^	1.24 (0.98–1.56)	5.1 ± 2.3	6.3 ± 2.9	0.083^+^	1.12 (0.83–1.52)
After discharge												
Discharge destination (home)	**42 (84%)**	**19 (53%)**	**0.002**^#^		36 (78%)	19 (63%)	0.155^#^		32 (74%)	10 (59%)	0.235^#^	
ER access or hospitalization	13 (26%)	13 (36%)	0.314^#^		14 (30%)	14 (47%)	0.152^#^		12 (28%)	6 (35%)	0.574^#^	

Bold indicates statistically significant results

*mRS* modified Rankin Scale, *ER* emergency room, +Independent sample t test, ^#^ Pearson’s chi-squared test, °Wilcoxon-Mann–Whitney U test, *Multivariate logistic regression models adjusted for age, living condition, cognitive impairment, pre-hospitalization mRS, length of hospitalization, and comorbidity sum score

**Table 5:** Factors associated with the survival outcome at 3, 6 and 12 months

	3 months follow-up				6 months follow-up				12 months follow-up			
	Survived	Deceased	p	Logistic regression*	Survived	Deceased	p	Logistic regression*	Survived	**Deceased**	p	Logistic regression*
	n = 86	n = 21		OR (95%CI)	n = 76	n = 8		OR (95%CI)	n = 60	n = 12		OR (95%CI)
At baseline												
Age	88.4 ± 2.8	89.9 ± 4	0.130^+^	1.14 (0.96–1.35)	88.5 ± 2.7	88.2 ± 4.2	0.844^+^	0.92 (0.676–1.29)	88.4 ± 2.6	89 ± 3.3	0.516^+^	1.11 (0.86–1.43)
Sex (male)	26 (30%)	4 (19%)	0.306^#^		22 (29%)	2 (25%)	0.814^#^		15 (25%)	6 (50%)	0.082^#^	
Coming from home	83 (96%)	21 (100%)	0.385^#^		74 (97%)	7 (87.5%)	0.153^#^		58 (97%)	12 (100%)	0.521^#^	
Living condition (alone)	35 (41%)	6 (32%)	0.439^#^	1.39 (0.35–5.55)	31 (41%)	3 (43%)	0.915^#^	2.22 (0.27–18.07)	26 (43%)	2 (17%)	0.084^#^	0.24 (0.04–1.48)
Cognitive impairment	34 (42%)	12 (57%)	0.213^#^	0.77 (0.19–3.17)	31 (44%)	2 (25%)	0.310^#^	**11.17 (1.06–117.40)**	23 (40%)	6 (54.5%)	0.383^#^	0.47 (0.09–2.32)
Pre-hospitalization mRS	**2 (0, 3)**	**4 (0.25, 4)**	**0.013°**	**1.53 (1.02–2.31)**	1 (0, 3)	3 (0.5, 4)	0.126°	1.75 (0.97–3.17)	2 (0, 3)	1 (0, 3)	0.300°	0.59 (0.33–1.07)
Length of hospitalization	8.5 (4.75, 12.25)	12 (4, 19)	0.231°	1.05 (0.99–1.11)	**7 (4, 12)**	**13.5 (9.25, 15.5)**	**0.026°**	1.04 (0.94–1.14)	7 (4, 11.75)	10 (4.75, 16)	0.269°	1.01 (0.93–1.10)
Discharge diagnosis (cerebrovascular event)	59 (69%)	13 (62%)	0.557^#^		52 (68%)	5 (62.5%)	0.733^#^		40 (67%)	9 (75%)	0.572^#^	
Comorbidity (sum score)	5.7 ± 2.8	5.9 ± 2.4	0.755^+^	0.83 (0.65–1.05)	**5.4 ± 2.7**	**8 ± 2.2**	**0.013**^+^	**1.64 (1.07–2.52)**	5.4 ± 2.5	5.7 ± 3.5	0.841^+^	1.02 (0.78–1.33)
After discharge												
Discharge destination (home)	61 (71%)	11 (65%)	0.609^#^		55 (72%)	5 (62.5%)	0.557^#^		42 (70%)	10 (83%)	0.347^#^	
ER access or hospitalization	–	–	–	–	–	–	–	–	–	–	–	

Bold indicates statistically significant results

*mRS* modified Rankin Scale, *ER* emergency room, ^+^ independent sample t test, ^#^Pearson’s chi-squared test, °Wilcoxon–Mann–Whitney U test, *Multivariate logistic regression models adjusted for age, living condition, cognitive impairment, pre-hospitalization mRS, length of hospitalization, and comorbidity sum score

Taking into account the results of univariate analyses, living condition, cognitive impairment, length of hospitalization, and comorbidity sum score were included as predictors in multivariate logistic regression models, that were further adjusted also for age and pre-hospitalization mRS (except for subgroup analyses on patients with pre-hospitalization mRS ≤ 3).

At 3 months, pre-hospitalization mRS (OR = 1.83, 95% CI 1.22–2.74), and living condition (OR = 0.30, 95% CI 0.09–0.99) were confirmed as significantly associated with the functional outcome (Table 4). In multivariate models limited to stroke patients, pre-hospitalization mRS (OR = 3.99, 95% CI 1.63–9.83) was confirmed as associated with the functional outcome together with the NIHSS at admission (OR = 1.37, 95% CI 1.11–1.69), while length of hospitalization was the only significant predictor in patients with pre-hospitalization mRS ≤ 3 (OR = 1.13, 95% CI 1.01–1.26) (supplementary tables 2 and 5).

Pre-hospitalization mRS resulted as the only factor significantly associated with the functional outcome both at 6 (OR = 1.57, 95% CI 1.06–2.34) and 12 months (OR = 1.62, 95% CI 1.05–2.72) (Table 4). Pre-hospitalization mRS (OR = 2.03, 95% CI 1.19–4.44) and NIHSS at admission (OR = 1.22, 95% CI 1.04–1.43) were associated with the functional outcome in stroke patients at 6 months, while no statistically significant predictors were found at 12 months (supplementary table 2). Pre-existing cognitive impairment resulted as the only significant predictor of the 12-month functional outcome in patients with pre-hospitalization mRS ≤ 3 (OR = 0.18, 95% CI 0.04-0.91) (supplementary table 5).

For the survival outcome, pre-hospitalization mRS was the only significant predictor at 3 months (OR = 1.53, 95% CI 1.02–2.31), while pre-existing cognitive impairment (OR = 11.17, 95% CI 1.06–117.40) and comorbidity sum score (OR = 1.64, 95% CI 1.07–2.52) were both confirmed as independent predictors at 6 months (Table 5). Subgroup analyses on predictors of the survival outcome at 3 months showed that only NIHSS at admission was relevant in stroke patients (OR = 1.33, 95% CI 1.10–1.61), and length of hospitalization was confirmed in patients with pre-hospitalization mRS ≤ 3 (OR = 1.14, 95% CI 1.01–1.29) (supplementary tables 3 and 6).

The multivariate models for the survival outcome at 12 months resulted in no statistically significant association, as in univariate analyses (Table 5).

## Discussion

We found that 1-year mortality of elderly patients admitted to a neurology unit for an acute neurological disease is extremely high. In parallel with mortality, also the impact on disability is huge in this frail population, with less than one fourth of patients left with only mild-to-moderate disability after 12 months. Despite these data were somehow expected, they might be of relevance for example as far as planning of future resources is concerned. Our study did not have the power to explore the effect of the specific reason for hospitalization on these data, although, as expected in this acute population, about two thirds of patients were admitted for an acute cerebrovascular event. Beyond the expected association between pre-hospitalization functional status and medium/long-term disability, other pre-existing contributing conditions, such as cognitive impairment and comorbidities, seemed to confirm the impact of clinical complexity on prognosis in the elderly.

Data on 1-year mortality rates in elderly were obtained from the Italian Mortality Database (IMDB) collected by the Italian National Institute of Statistics (ISTAT). In 2020, ISTAT data reported the following mortality rates per 1000 individuals per year: 258 in the age group 85–89, 447 in the group 90–94, and 672 > 95 years. Stratifying our data according to the same age ranges, our patients presented higher mortality rates both in the 85–89 group (342 per 1000 individuals per year) and > 95 (857 per 1000 individuals per year), with an increased probability of death of approximately 30%.

Looking at hospitalized elderly patients, our results on survival outcome are in line with data from two previous studies including very elderly patients admitted to general hospital for acute illness or exacerbation of a chronic disease [7, 8]. In an Italian retrospective observational study including 529 patients (mean age 84.6 ± 7.3 years) admitted to a Geriatric Unit from the Emergency Department, Zanetti and colleagues found a 1-year mortality rate of 36% [7]. Furthermore, the authors found that male gender had a significantly higher mortality rate compared to the female one at 1 year, and a similar trend emerged in our cohort at 12 months. These results are consistent with evidence coming from a meta-analysis showing that mortality of older adults is sex-dependent, with males having higher mortality risk than females, independently of frailty [9]. A study by Garåsen and colleagues found a mortality rate of 31% in a sample of elderly patients (mean age 81.3 ± 0.8 years) admitted to a general hospital [8]. Interestingly, in the above study the 1-year mortality rate decreased at 18% when patients were referred to an intermediate care at a community hospital, i.e., a patient-focused program based on the combination of treatment of the diseases and supportive services aimed at maximizing patients’ and families’ knowledge and control. In another study focused on survival rates after acute stroke, Magdon-Ismail and colleagues found a 1-year mortality rate of approximately 8% in a younger cohort (mean age 68.6 ± 14.8 years) [10].

Our study has several limitations. The first one is that the sample size is limited, partly because of the occurrence of the COVID-19 pandemics during its progression that prevented additional enrolment in our unit that was shut down during that period. However, we believe that the results we reported are of interest as they shoot the picture of a neurology general ward for acute patients. Also, the follow up was exclusively done by phone interviews although we focused on strong outcomes such as mortality and basic functional outcome that can be reliably assessed in this way. Other outcomes, for example cognitive measures, might be of interest in defining the outcome of this very old population. It should be noted however that a great amount of our patients had already pre-existing cognitive impairment, which of course is a strong predictor of poor outcome. The high incidence of pre-existing cognitive decline is consistent with previous data from the same area [11]. At present, we have no information about whether a reduced compliance to treatment in this very old population might be, together with others, a reason for the poor outcome. Another limitation of our study is the lack of a control group and of younger age-groups. Finally, our possibilities to compare our results with previous data on mortality and functional outcomes for the elderly, with or without hospitalization, are very limited. From one side, there is paucity of data on medium-term outcomes in elderly patients dismissed from internal medicine units, and specifically, from neurology departments. On the other side, in the literature ‘elderly’ samples typically include patients with age varying from 60 to 85 years, while no study used a cut-off of 85 years, and very few specific data are available for the oldest ones.

In conclusion, hospitalization of patients older than 85 years for acute neurological conditions is associated with high mortality and great disability burden. These results have to be confirmed in larger studies. Our study was not set, nor has the power, to explore and deliver information about the use of medical resources in this elderly population. However, it might be a first step on the way to produce such type of data.

## Supplementary Information

Below is the link to the electronic supplementary material.

Supplementary file1 (DOCX 49 KB)

## Data Availability

The datasets generated during and/or analyzed during the current study are available from the corresponding author on reasonable request.
