# Supplementary material for: Outcome of patients older than 85 years hospitalized in a neurology unit
Source: Aging Clin Exp Res. 2023 Jun 18;35(8):1753–61. doi: 10.1007/s40520-023-02468-x (PMC10363031; doi:10.1007/s40520-023-02468-x)
Supplement: Supplementary file 1 — Supplementary file1 (DOCX 49 KB) [file 40520_2023_2468_MOESM1_ESM.docx]

**Aging Clinical and Experimental Research**

**Outcome of patients older than 85 years hospitalized in a neurology unit**

Giacomo Querzola,^1^* Andrea Bellomo,^1^* Emilia Salvadori,^2^* Leonardo Pantoni^3-4^

^1^ Department of Biomedical and Clinical Sciences, University of Milan, Milan, Italy

^2^ NEUROFARBA Department, Neuroscience Section, University of Florence, Florence, Italy

^3^ Neurology Unit, Luigi Sacco University Hospital, Milan, Italy

^4^ Stroke and Dementia Lab, “Luigi Sacco” Department of Biomedical and Clinical Sciences, University of Milan, Milan, Italy

* These authors contributed equally to this work

**Corresponding author:**

Leonardo Pantoni, MD, PhD

Department of Biomedical and Clinical Sciences

University of Milan

Via Giovanni Battista Grassi, 74, 20157 Milano, Italy
Phone: (+39) 02 3904 2317

E-mail: [leonardo.pantoni@unimi.it](mailto:leonardo.pantoni@unimi.it)

ORCID: [0000-0001-7357-8530](https://orcid.org/0000-0001-7357-8530)

**Supplementary Table 1.** Characteristics of stroke patients seen at the follow-up at 3, 6 and 12 months.

|  |  | **3 months follow-up** | | **6 months follow-up** | | **12 months follow-up** | |
| --- | --- | --- | --- | --- | --- | --- | --- |
|  |  | n=72 |  | n=57 |  | n=72 |  |
| **At baseline** | Age | 72 | 88.9±3.1 | 57 | 88.6±2.7 | 49 | 88.9±2.8 |
|  | Sex (male) | 72 | 20 (28%) | 57 | 16 (28%) | 49 | 14 (29%) |
|  | Coming from home | 72 | 70 (97%) | 57 | 55 (96%) | 49 | 48 (98%) |
|  | Living condition (alone) | 72 | 25 (35%) | 57 | 21 (37%) | 49 | 18 (37%) |
|  | Cognitive impairment | 69 | 33 (48%) | 54 | 25 (46%) | 46 | 22 (48%) |
|  | Pre-hospitalization mRS | 69 | 2 (0, 3) | 55 | 1 (0, 3) | 47 | 1 (0, 3) |
|  | NIHSS | 72 | 8.4±7.6 | 57 | 6.6±6.1 | 49 | 5.9±5.5 |
|  | Length of hospitalization | 70 | 9 (5, 13.25) | 57 | 9 (5, 13.5) | 49 | 8 (5, 13) |
|  | Comorbidity (sum score) | 72 | 5.9±2.9 | 57 | 5.8±3 | 49 | 5.5±3 |
| **After discharge** | Discharge destination (home) | 68 | 41 (60%) | 57 | 35 (61%) | 49 | 31 (63%) |
|  | ER access or hospitalization | 72 | 15 (25%) | 52 | 20 (38%) | 40 | 10 (25%) |

mRS: modified Rankin Scale; NIHSS: National Institutes of Health Stroke Scale; ER: Emergency Room

**Supplementary Table 2.** Subgroup analysis on stroke patients: factors associated with the functional outcome at 3, 6 and 12 months.

|  |  | **3 months follow-up** | | | | **6 months follow-up** | | | | **12 months follow-up** | | | |
| --- | --- | --- | --- | --- | --- | --- | --- | --- | --- | --- | --- | --- | --- |
|  |  | **mRS≤3** | **3<mRS<6** |  | *Logistic regression** | **mRS≤3** | **3<mRS<6** |  | *Logistic regression** | **mRS≤3** | **3<mRS<6** |  | *Logistic regression** |
|  |  | n=31 | n=28 | p | OR (95%CI) | n=28 | n=24 | p | OR (95%CI) | n=26 | n=14 | p | OR (95%CI) |
| **At baseline** | Age | 88.4±2.7 | 88.8±2.8 | .577**^+^** | 1.21  (.84-1.75) | 88.5±2.5 | 88.3±2.9 | .283^+^ | 1.09  (.84-1.41) | 88.6±2.4 | 88.7±3.2 | .881^+^ | .96  (.69-1.31) |
|  | Sex (male) | 11 (35%) | 7 (25%) | .382^#^ |  | 9 (32%) | 6 (25%) | .571^#^ |  | 9 (35%) | 1 (7%) | .056^#^ |  |
|  | Coming from home | 30 (97%) | 27 (96%) | .942^#^ |  | 27 (96%) | 24 (100%) | .350^#^ |  | 25 (96%) | 14 (100%) | .457^#^ |  |
|  | Living condition (alone) | **16 (52%)** | **6 (21%)** | **.017**^#^ | .30  (.05-1.87) | 14 (50%) | 18 (75%) | .065^#^ | .39  (.08-1.85) | 12 (46%) | 4 (29%) | .279^#^ | .85  (.14-5.34) |
|  | Cognitive impairment | 10 (34%) | 16 (59%) | .063^#^ | 3.49  (.46-26.33) | 10 (38%) | 14 (61%) | .117^#^ | 2.73  (.44-16.91) | **7 (29%)** | **10 (71%)** | **.011**^#^ | .39  (.06-2.58) |
|  | Pre-hospitalization mRS | **1 (0, 2)** | **3 (1, 4)** | **.001°** | **3.99**  **(1.63-9.83)** | 1 (0, 2) | 3 (0, 4) | **.010**° | **2.03**  **(1.19-4.44)** | **1 (0, 2.5)** | **3 (1, 4)** | **.017**° | 1.83  (.92-3.65) |
|  | NIHSS | **3.9±3.9** | **9.5±6.8** | **.001^+^** | **1.37**  **(1.11-1.69)** | **4.1±3.9** | **8.1±6.5** | **.007^+^** | **1.22**  **(1.04-1.43)** | **3.8±4.1** | **7.8±5.6** | **.008^+^** | 1.29  (1.02-1.64) |
|  | Length of hospitalization | 8 (4, 12) | 10 (6.25, 18.25) | .052° | 1.07  (.89-1.29) | 8 (3.5, 12) | 9.5 (5.25, 13.75) | .387° | .97  (.87-1.08) | 7 (4.75, 12) | 9.5 (6.75, 15.75) | .180° | 1.01  (.89-1.12) |
|  | Comorbidity (sum score) | **4.8±2.6** | **7.1±3.1** | **.003^+^** | 1.18  (.86-1.62) | **4.6±2.5** | **6.7±3.2** | **.012**^+^ | 1.21  (.91-1.62) | 5±2.7 | 6.6±3.1 | .111^+^ | .90  (.62-1.32) |
| **After discharge** | Discharge destination (home) | **23 (74%)** | **13 (46%)** | **.029**^#^ |  | 19 (68%) | 14 (58%) | .477^#^ |  | 16 (61%) | 8 (57%) | .787^#^ |  |
|  | ER access or hospitalization | 7 (23%) | 8 (29%) | .598^#^ |  | 9 (32%) | 11 (46%) | .312^#^ |  | 5 (19%) | 5 (36%) | .251^#^ |  |

mRS: modified Rankin Scale; NIHSS: National Institutes of Health Stroke Scale; ER: Emergency Room

^+^ Independent sample t test, ^#^ Pearson's chi-squared test, ° Wilcoxon-Mann-Whitney U test, * Multivariate logistic regression models adjusted for age, living condition, cognitive impairment, pre-hospitalization mRS, NIHSS, length of hospitalization, and comorbidity sum score

**Supplementary Table 3.** Subgroup analysis on stroke patients: factors associated with the survival outcome at 3, 6 and 12 months.

|  |  | **3 months follow-up** | | | | **6 months follow-up** | | | | **12 months follow-up** | | | |
| --- | --- | --- | --- | --- | --- | --- | --- | --- | --- | --- | --- | --- | --- |
|  |  | **Survived** | **Deceased** |  | *Logistic regression** | **Survived** | **Deceased** |  | *Logistic regression** | **Survived** | **Deceased** |  | *Logistic regression** |
|  |  | n=59 | n=13 | p | OR (95%CI) | n=52 | n=5 | p | OR (95%CI) | n=40 | n=9 | p | OR (95%CI) |
| **At baseline** | Age | **88.6±2.7** | **90.8±3.9** | **.018^+^** | 1.36  (.95-1.95) | 88.8±2.7 | 86.6±1.5 | .079^+^ | .54  (.28-1.05) | 88.6±2.7 | 90.1±3.1 | .152^+^ | 1.22  (.90-1.64) |
|  | Sex (male) | 18 (30%) | 2 (15%) | .270^#^ |  | 15 (29%) | 1 (20%) | .674^#^ |  | 10 (25%) | 4 (44%) | .243^#^ |  |
|  | Coming from home | 57 (97%) | 13 (100%) | .501^#^ |  | **51 (98%)** | **4 (80%)** | **.036**^#^ |  | 39 (97%) | 9 (100%) | .632^#^ |  |
|  | Living condition (alone) | 22 (37%) | 3 (23%) | .330^#^ | .47  (.03-8.52) | 20 (38%) | 1 (20%) | .414^#^ | - | 16 (40%) | 2 (22%) | .318^#^ | .29  (.03-2.53) |
|  | Cognitive impairment | 26 (46%) | 7 (54%) | .630^#^ | .95  (.03-28.81) | 24 (49%) | 1 (20%) | .216^#^ | - | 17 (45%) | 5 (62%) | .361^#^ | .19  (.02-1.99) |
|  | Pre-hospitalization mRS | **1 (0, 3)** | **3.5 (1, 4.75)** | **.031°** | 1.55  (.66-3.67) | 1 (0, 3) | 3 (1, 4) | .232° | 1.57  (.62-3.97) | 1 (0, 3) | 1 (0, 3) | .647° | .64  (.29-1.38) |
|  | NIHSS | **6.5±6.1** | **16.8±8.2** | **.001^+^** | **1.33**  **(1.10-1.61)** | **5.9±5.6** | **12.8±8.2** | **.016^+^** | 1.21  (.94-1.57) | **5.2±5** | **9±6.7** | **.031^+^** | 1.15  (.97-1.37) |
|  | Length of hospitalization | 9 (5, 14) | 7 (4, 12) | .528**°** | .98  (.89-1.06) | 8.5 (5, 12.75) | 10 (7.5, 21.5) | .327° | .95  (.83-1.09) | 8 (5, 12) | 10 (3.5, 17) | .889° | .95  (.86-1.06) |
|  | Comorbidity (sum score) | 5.9±3 | 6.1±2.4 | .805**^+^** | .67  (.40-1.12) | **5.6±3** | **8.8±1.9** | **.001**^+^ | 1.72  (.70-4.21) | 5.6±2.9 | 5.3±3.7 | .831^+^ | .91  (.66-1.24) |
| **After discharge** | Discharge destination (home) | 36 (61%) | 5 (56%) | .755^#^ |  | 33 (63%) | 2 (40%) | .303^#^ |  | 24 (60%) | 7 (78%) | .318^#^ |  |
|  | ER access or hospitalization | - | - | - |  | - | - | - |  | - | - | - |  |

mRS: modified Rankin Scale; NIHSS: National Institutes of Health Stroke Scale; ER: Emergency Room

^+^ Independent sample t test, ^#^ Pearson's chi-squared test, ° Wilcoxon-Mann-Whitney U test, * Multivariate logistic regression models adjusted for age, living condition, cognitive impairment, pre-hospitalization mRS, NIHSS, length of hospitalization, and comorbidity sum score

**Supplementary Table 4.** Characteristics of patients with pre-hospitalization mRS≤3 seen at the follow-up at 3, 6 and 12 months.

|  |  | **3 months follow-up** | | **6 months follow-up** | | **12 months follow-up** | |
| --- | --- | --- | --- | --- | --- | --- | --- |
|  |  | n=81 |  | n=70 |  | n=61 |  |
| **At baseline** | Age | 81 | 88.2±2.6 | 70 | 88.2±2.6 | 61 | 88.4±2.7 |
|  | Sex (male) | 81 | 26 (32%) | 70 | 22 (31%) | 61 | 19 (31%) |
|  | Coming from home | 81 | 80 (99%) | 70 | 69 (99%) | 61 | 60 (98%) |
|  | Living condition (alone) | 81 | 39 (48%) | 70 | 32 (46%) | 61 | 26 (43%) |
|  | Cognitive impairment | 78 | 27 (35%) | 67 | 25 (37%) | 59 | 23 (39%) |
|  | Length of hospitalization | 81 | 9 (4, 14) | 70 | 8.5 (4, 12.25) | 61 | 8 (4, 12) |
|  | Discharge diagnosis (cerebrovascular event) | 81 | 53 (65%) | 70 | 45 (64%) | 61 | 39 (64%) |
|  | Comorbidity (sum score) | 81 | 5.6±2.6 | 70 | 5.6±2.6 | 61 | 5.5±2.5 |
| **After discharge** | Discharge destination (home) | 79 | 53 (67%) | 70 | 50 (71%) | 61 | 44 (72%) |
|  | ER access or hospitalization | 72 | 23 (32%) | 65 | 23 (35%) | 50 | 16 (32%) |

mRS: modified Rankin Scale; ER: Emergency Room

**Supplementary Table 5.** Subgroup analysis on patients with pre-hospitalization mRS≤3: factors associated with the functional outcome at 3, 6 and 12 months.

|  |  | **3 months follow-up** | | | | **6 months follow-up** | | | | **12 months follow-up** | | | |
| --- | --- | --- | --- | --- | --- | --- | --- | --- | --- | --- | --- | --- | --- |
|  |  | **mRS≤3** | **3<mRS<6** |  | *Logistic regression** | **mRS≤3** | **3<mRS<6** |  | *Logistic regression** | **mRS≤3** | **3<mRS<6** |  | *Logistic regression** |
|  |  | n=49 | n=23 | p | OR (95%CI) | n=44 | n=21 | p | OR (95%CI) | n=39 | n=11 | p | OR (95%CI) |
| **At baseline** | Age | 88.2±2.6 | 88.2±2.6 | .494**^+^** | 1.09  (.85-1.38) | 88.1±2.5 | 88.8±2.9 | .300^+^ | 1.10  (.88-1.38) | 88.2±2.5 | 88.4±2.8 | .274^+^ | .92  (.67-1.27) |
|  | Sex (male) | 15 (31%) | 9 (39%) | .475^#^ |  | 14 (32%) | 6 (29%) | .791^#^ |  | 12 (31%) | 1 (9%) | .148^#^ |  |
|  | Coming from home | 48 (98%) | 23 (100%) | .490^#^ |  | 43 (98%) | 21 (100%) | .486^#^ |  | 38 (97%) | 11 (100%) | .592^#^ |  |
|  | Living condition (alone) | **28 (57%)** | **5 (22%)** | **.005**^#^ | .29  (.08-1.01) | 23 (52%) | 15 (71%) | .072^#^ | .44  (.13-1.51) | 19 (49%) | 5 (45%) | .848^#^ | 1.61  (.32-8.02) |
|  | Cognitive impairment | 15 (33%) | 11 (48%) | .219^#^ | 1.11  (.32-3.89) | 14 (34%) | 11 (52%) | .166^#^ | .75  (.22-2.52) | **10 (27%)** | **7 (64%)** | **.026**^#^ | **.18**  **(.04-.91)** |
|  | Length of hospitalization | **7 (4, 11.5)** | **11 (7, 19)** | **.005°** | **1.13**  **(1.01-1.26)** | 7 (4, 11.75) | 10 (4.5, 13.5) | .226° | 1.01  (.91-1.12) | 7 (4, 12) | 8 (6, 11) | .488° | .96  (.85-1.09) |
|  | Discharge diagnosis (cerebrovascular event) | 30 (61%) | 17 (74%) | .292^#^ |  | 26 (59%) | 16 (76%) | .178^#^ |  | 22 (56%) | 9 (82%) | .125 |  |
|  | Comorbidity (sum score) | **5.1±2.3** | **6.9±2.8** | **.002^+^** | 1.18  (.93-1.51) | **4.9±2.2** | **6.5±3** | **.022**^+^ | 1.17  (.92-1.50) | 5.1±2.1 | 6.2±2.9 | .777^+^ | 1.28  (.87-1.87) |
| **After discharge** | Discharge destination (home) | **41 (84%)** | **10 (43%)** | **.001**^#^ |  | 35 (79%) | 12 (57%) | .059^#^ |  | **30 (77%)** | **5 (45%)** | **.044^#^** |  |
|  | ER access or hospitalization | 13 (26%) | 10 (43%) | .150^#^ |  | 13 (29%) | 10 (48%) | .154^#^ |  | 10 (26%) | 6 (54%) | .070^#^ |  |

mRS: modified Rankin Scale; ER: Emergency Room

^+^ Independent sample t test, ^#^ Pearson's chi-squared test, ° Wilcoxon-Mann-Whitney U test,

* Multivariate logistic regression models adjusted for age, living condition, cognitive impairment, length of hospitalization, and comorbidity sum score

**Supplementary Table 6.** Subgroup analysis on patients with pre-hospitalization mRS≤3: factors associated with the survival outcome at 3, 6 and 12 months.

|  |  | **3 months follow-up** | | | | **6 months follow-up** | | | | **12 months follow-up** | | | |
| --- | --- | --- | --- | --- | --- | --- | --- | --- | --- | --- | --- | --- | --- |
|  |  | **Survived** | **Deceased** |  | *Logistic regression** | **Survived** | **Deceased** |  | *Logistic regression** | **Survived** | **Deceased** |  | *Logistic regression** |
|  |  | n=72 | n=9 | p | OR (95%CI) | n=65 | n=5 | p | OR (95%CI) | n=50 | n=11 | p | OR (95%CI) |
| **At baseline** | Age | 88.2±2.6 | 88.2±2.5 | .964^+^ | 1.01  (.71-1.42) | 88.3±2.7 | 87.2±1.8 | .367^+^ | .84  (.52-1.36) | 88.3±2.5 | 88.9±3.4 | .476^+^ | 1.17  (.88-1.55) |
|  | Sex (male) | 24 (33%) | 2 (22%) | .501^#^ |  | 20 (31%) | 2 (40%) | .668^#^ |  | 13 (26%) | 6 (54%) | .064^#^ |  |
|  | Coming from home | 71 (99%) | 9 (100%) | .722^#^ |  | 64 (98%) | 5 (100%) | .780^#^ |  | 49 (98%) | 11 (100%) | .636^#^ |  |
|  | Living condition (alone) | 33 (46%) | 6 (67%) | .238^#^ | 3.52  (.53-23.45) | 29 (45%) | 3 (60%) | .506^#^ | 14.63  (.70-303.72) | 24 (48%) | 2 (18%) | .070^#^ | .24  (.04-1.40) |
|  | Cognitive impairment | 26 (38%) | 1 (11%) | .115^#^ | 2.59  (.24-28.30) | 25 (40%) | 0 (0%) | .073^#^ | - | 17 (35%) | 6 (54%) | .241^#^ | .73  (.16-3.32) |
|  | Length of hospitalization | 9  (4, 12.75) | 16.5  (7, 19.75) | .090**°** | **1.14**  **(1.01-1.29)** | 8  (4, 12) | 14  (11, 21.5) | **.017°** | 1.19  (.99-1.42) | 7  (4, 11.25) | 10  (4, 16) | .204° | 1.06  (.94-1.19) |
|  | Discharge diagnosis (cerebrovascular event) | 47 (65%) | 6 (67%) | .934^#^ |  | 42 (65%) | 3 (60%) | .836^#^ |  | 31 (62%) | 8 (73%) | .502^#^ |  |
|  | Comorbidity (sum score) | 5.6±2.6 | 5.1±2.5 | .559**^+^** | .83  (.57-1.21) | 5.4±2.6 | 7.4±2.7 | .105^+^ | 1.51  (.85-2.69) | 5.3±2.3 | 6.2±3.2 | .312^+^ | .99  (.74-1.34) |
| **After discharge** | Discharge destination (home) | **51 (71%)** | **2 (29%)** | **.023^#^** |  | 47 (72%) | 3 (60%) | .557^#^ |  | 35 (70%) | 9 (82%) | .429^#^ |  |
|  | ER access or hospitalization | - | - | - |  | - | - | - |  | - | - | - |  |

mRS: modified Rankin Scale; ER: Emergency Room

^+^ Independent sample t test, ^#^ Pearson's chi-squared test, ° Wilcoxon-Mann-Whitney U test,

* Multivariate logistic regression models adjusted for age, living condition, cognitive impairment, length of hospitalization, and comorbidity sum score
